# Impaired Spatial Learning and Memory after Sevoflurane–Nitrous Oxide Anesthesia in Aged Rats Is Associated with Down-Regulated cAMP/CREB Signaling

**DOI:** 10.1371/journal.pone.0079408

**Published:** 2013-11-15

**Authors:** Wan-Xia Xiong, Guo-Xia Zhou, Bei Wang, Zhang-Gang Xue, Lu Wang, Hui-Chuan Sun, Sheng-Jin Ge

**Affiliations:** 1 Department of Anesthesia, Zhongshan Hospital and Department of Anesthesiology, Shanghai Medical College, Fudan University, Shanghai, China; 2 Department of Anesthesia, Shanghai Fifth People’s Hospital, Fudan University, Shanghai, China; 3 Liver Cancer Institute and Zhongshan Hospital, Fudan University, Shanghai, China; Universidade do Estado do Rio de Janeiro, Brazil

## Abstract

Neurocognitive deficits arising from anesthetic exposure have recently been debated, while studies have shown that the phosphorylation of cyclic AMP response element-binding protein (CREB) in the hippocampus is critical for long-term memory. To better understand the neural effects of inhalational anesthetics, we studied the behavioral and biochemical changes in aged rats that were exposed to sevoflurane (Sev) and nitrous oxide (N_2_O) for 4 h. Eighteen-month-old rats were randomly assigned to receive 1.3% sevoflurane and 50% nitrous oxide/50% oxygen or 50% oxygen for 4 h. Spatial learning and memory were tested with the Morris water maze 48 h after exposure, and the results showed that sevoflurane–nitrous oxide exposure induced a significant deficit in spatial learning acquisition and memory retention. Experiments revealed that the cAMP and pCREB levels in the dorsal hippocampus were decreased in rats with anesthetic exposure in comparison with control rats 48 h after anesthesia as well as 15 min after the probe trial, but there were no significant differences in CREB expression. Besides these, the current study also found the DG neurogenesis significantly decreased as well as neuronal loss and neuronal apoptosis increased in the hippocampus of rats exposed to Sev+N_2_O. The current study demonstrated that down-regulation of cAMP/CREB signaling, decrease of CREB-dependent neurogenesis and neuronal survival in the hippocampus contributed to the neurotoxicity and cognitive dysfunction induced by general anesthesia with sevoflurane–nitrous oxide.

## Introduction

The combination of sevoflurane with nitrous oxide is widely used in clinical anesthesia practice. However, recent studies have raised concerns about the neurotoxicity of inhalational anesthetics and their contribution to postoperative cognitive dysfunction (POCD) [1], [2], [3]. Studies indicated that general anesthesia with a combination of nitrous oxide (N_2_O) and isoflurane (ISO) or sedation with 70% N_2_O produced lasting impairment in spatial working memory in rats [4], [5], [6], [7], [8]. Exposure of neonatal mice to inhaled sevoflurane not only caused persistent learning deficits in fear conditioning later in adulthood, but also abnormal social behaviors resembling autism spectrum disorder [9]. In addition, such exposure induced apoptosis, increased beta-amyloid protein levels [1] and tau phosphorylation through activation of specific kinases, which is considered a potential mechanism of cognitive dysfunction caused by anesthesia [10]. Although detrimental effects of anesthetics on cognitive function have been reported, to our knowledge, no study has investigated the effects of the anesthetic sevoflurane combined with N_2_O on spatial learning and memory in aged rats.

A current “hot spot” of memory research involves the cyclic AMP response element-binding protein (CREB), which has been extensively implicated in learning and memory [11], long term potentiation(LTP) [12], and neuroprotection [13]. It is fairly well established that hippocampus-mediated memory consolidation involves signaling cascades leading to gene transcription of the transcription factor CREB [14]. Phosphorylation/activation of CREB (pCREB) on Ser 133 by cyclic AMP- or Ca^2+^-dependent protein kinase is critical for long-term memory consolidation [15], [16], [17]. Inhibition of phosphodiesterase-4 (PDE4), an enzyme that catalyzes cAMP hydrolysis, increases cAMP and phosphorylation of CREB [18], [19], facilitates induction of hippocampal LTP [20] and enhances memory [21], [22]. Consistent with this, several studies have shown that pCREB is also involved in hippocampal neurogenesis, influences the neurotrophic factor-dependent survival of culture neurons and regulates several steps of neurogenesis including proliferation, differentiation, and survival [23], [24], [25]. To our knowledge, adult neurogenesis in the hippocampus plays a key role in spatial memory function, regulating acquisition of a spatial memory and the subsequent flexible use of spatially precise learning strategies [26], [27], [28]. Besides the neurogenesis, CREB phosphorylation has also been found to be important in the neurotrophin-mediated neuronal survival [29], [30]. Studies showed that ablation of neuronal CREB during development resulted in a massive neuronal apoptosis, and a full CREB-KO mice showed a significant increase in neuronal cell death in dorsal root ganglion neurons [31], [32]. Based on these, inhibition of cAMP/CREB induced by anesthetics would lead to the decrease of neurogenesis but increase of neuronal cell death, and further aggravated cognitive dysfunctions.

The aim of the present study is to determine whether anesthesia with sevoflurane combined with N_2_O in aged rats could induce spatial learning and memory deficit. We also evaluated the cAMP/CREB signaling, neurogenesis levels and cell survival in the hippocampus in an effort to test the hypothesis that general anesthesia by Sev+N_2_O down-regulates cAMP/CREB pathway, and then suppresses neuronal survival and hippocampal DG neurogenesis, subsequently aggravating learning and memory deficit.

## Materials and Methods

### Animals

The experimental protocol was approved by the Shanghai Medical Experimental Animal Care Commission. Male Sprague Dawley rats were obtained from Shanghai Laboratory Animal Center of the Chinese Academy of Sciences. Aged rats (18 months old) were housed one or two per cage in a climate- and humidity-controlled room in the animal facilities on a 12-h light–dark artificial cycle (lights on at 7∶00 AM) with free access to food and water. All experiments were performed during the light phase between 7∶00 AM and 7∶00 PM.

### Anesthesia Procedure

Animals (*n* = 40) were randomized into four groups (10 in each group): Sev+N_2_O-MWM, in which rats received sevoflurane-N_2_O and behavioral training(Morris Water Maze, MWM); Sev+N_2_O, in which rats received anesthesia without behavioral training; Con-MWM, in which rats received control gas and behavioral training; and Con, in which rats received control gas without behavioral training. Rats that were randomly assigned to the anesthesia groups received 1.3% sevoflurane (USP, Baxter, Deerfield, IL, USA) in 50% N_2_O/50% oxygen for 4 h at a flow rate of approximately 3 L/min in a Plexiglas anesthetizing chamber, which was adjusted to maintain *minimum* alveolar concentration, oxygen, and carbon dioxide at constant levels. Gases within the anesthetic chamber were monitored continuously, and arterial oxygen saturation was measured noninvasively using a pulse oximeter during anesthesia. Control groups received 50% oxygen in their home cage at identical flow rates as anesthetized animals for 4 h, but arterial oxygen saturation was not measured to prevent the introduction of stress as a confounding variable. All anesthetized rats were breathing spontaneously, and the temperature of the anesthetizing chamber was controlled to maintain rat temperature at 37°C ±0.5°C using a heating pad. Anesthesia was terminated by discontinuing the anesthetics. Rats were allowed to recover for 48 h to avoid the confounding influence of residual anesthetic. Forty-eight hours after anesthesia, rats in the groups without behavioral training were sacrificed, and the remaining rats were tested in the Morris water maze from day 1 to day 6.

### Morris Water Maze Task

Spatial memory ability was examined in a water maze that consisted of a swimming pool as described by Morris [33] and adapted for rats. It consisted of a circular tank (160-cm diameter, 50 cm high), filled to a depth of 30 cm with water maintained at 22°C and rendered opaque by the addition of white nontoxic paint. The pool was located in a room uniformly illuminated by a halogen lamp and equipped with various distal cues. Located inside the pool was a removable, circular (12-cm diameter) platform (PF) made of transparent Plexiglas, positioned such that its top surface was 1.0 cm below the surface of the water. The platform, which served as a refuge from the water, was generally located in the center of an arbitrarily defined quadrant of the maze. The quadrant where the PF was located was defined as the target quadrant (E quadrant), and the other three were defined as N, S, W quandrant. The swim paths of the animals were captured by a video camera mounted above the water maze, and the corresponding tracks were recorded and analyzed by a computer tracking system (VideoTrack, Viewpoint).

### Behavioral Procedures

During the learning procedure, rats were tested during the light phase between 7∶00 AM and 7∶00 PM. Each rat was given a daily four-trial session (30-min intertrial interval) for six consecutive days. Each trial consisted of releasing the rat into the water facing the outer edge of the pool at one of the quadrants (in a random sequence) and letting the animal escape to the submerged platform. Rats were allowed to swim for a maximum of 60 s in each trial, and the time they spent before reaching the platform (i.e., escape latency) was a measure of acquisition of spatial navigation. If the rat failed to find the platform within 60 s, it was manually guided to the platform, and the escape latency was accepted as 60 s. After climbing onto the platform, the rat was left on it for 15 s and then removed from the pool and returned to its cage beneath a heat lamp to reduce the drop in core temperature. The release point differed in each trial, and different sequences of release points were used from day to day.

Twenty-four hours following the acquisition phase, rats were subjected to a probe trial in which the platform was removed. Starting from the quadrant opposite from the target quadrant where the platform had been located, each rat was allowed to swim for 60 s. Time spent and distances covered in the four round probe zones were measured by the tracking system. Data were collected using the VideoTrack system.

### Protein Extraction from Hippocampal Tissue

Five rats in Sev+N_2_O-MWM group and six rats in Con-MWM group were deeply anesthetized at 15 min after the probe trial, the brains were immediately removed, and both hippocampi from each rat were dissected out. Hippocampi were homogenized by brief sonication in an extract buffer (400 µl) containing 20 mM Tris-HCl (pH 7.4), 150 mM NaCl, 1 mM EDTA, 1 mM EGTA, 1 mM PMSF, 10 µg/ml aprotinin, 1% Triton X-100, and phosphatase inhibitor cocktail 1 and 2 (Sigma, St. Louis, MO). The homogenate was centrifuged at 12,000 *g* for 10 min at 4°C. The supernatant was removed and stored at −80°C until use.

For the two groups without Morris water maze training, 48 h after Sev+N_2_O anesthesia or control, hippocampi from five rats per group were extracted and tissues were processed as already described.

### Western Blot

Samples (30 µg of protein) were subjected to SDS-PAGE (10% gels) and then transferred from the gel to nitrocellulose membranes using a tank transfer apparatus with buffer containing 25 mM bicine, 25 mM Bis-Tris, and 20% methanol. Membranes were incubated in blocking buffer (TBS/0.1% Tween 20 with 5% nonfat dried milk) for 2 h at room temperature. To control for protein loading, an antibody against β-actin (Santa Cruz Technologies, Santa Cruz, CA) was used. Anti-CREB antibody (1∶1000; Cell Signaling Technologies, Danvers, MA), anti-pCREB antibody (1∶1000; Cell Signaling Technologies, Danvers, MA) and anti-Bax (1∶1000; Santa Cruz Technologies, CA) were added to the buffer solution, and the membranes were incubated overnight at 4°C. The membrane was then washed for 10 min three times in 1× TBS/0.1% Tween 20, and then incubated in HRP-conjugated anti-rabbit IgG (secondary antibody, 1∶3000; Cell Signaling) in blocking buffer for 1 h. Subsequently, membranes were washed with 1× TBS/0.1% Tween 20 several times. Blots were visualized with an ECL detection kit (Pierce, IL) and analyzed using Quantity One 1-D Analysis Software (Bio-Rad, San Francisco, CA).

### Cyclic AMP Analysis

Cyclic AMP (cAMP) levels were determined using a cAMP Complete ELISA kit (Enzo Life Sciences, USA) according to the manufacturer’s instructions. The level of cAMP in the sample was determined based on a standard curve and expressed as pmol/mg per each sample.

### Immunohistochemical Procedures

The remaining rats in each group were deeply anesthetized and perfused transcardially with ice-cold phosphate-buffered solution (PBS) and 4% paraformaldehyde (in 0.1 M phosphate buffer, pH 7.4). The brains were then dissected out, and the meninges were carefully removed and then post-fixed in the same fixative overnight, cryoprotected by first sinking in 10% and then in 30% sucrose (in 0.1 M phosphate buffer) at 4°C. Coronal sections (20 µm) were cut on a microtome.

### Immunofluorescence

After blocking of nonspecific epitopes with PBS containing 10% donkey serum and 0.5% Triton-100 for 1 h at room temperature and then in the primary antibody : Phospho-CREB Rabbit mAb (1∶100, Cell Signaling), NeuN (1∶200, Millipore), five sections (20 µm thickness, 80 µm space) of each rat were incubated for 48 h at 4°C. Sections were then washed in PBS and incubated for 4 h with a 1∶1000 dilution of anti-rabbit or anti-mouse IgG secondary antibody (Invitrogen, Carlsbad, CA, USA). Nuclear counterstaining was performed with 4′,6-diamidino-2-phenylindole (DAPI, Sigma) for 30 min at room temperature before three washes with dH_2_O for 10 min each time and coverslipped. Quantitative analysis was performed using an imaging analysis system (Leica Qwin). Assessment of pCREB staining was performed in CA1 and CA3 area of the dorsal hippocampus, while assessment of NeuN-positive neurons was performed in the CA1 area of the dorsal hippocampus.

### Nissl Staining

Sections rinse in tap water and then in distilled water. Stain in 0.1% cresyl violet solution for 3–10 min. Rinse quickly in distilled water. Then differentiate and dehydrate in alcohol then clearing and finally mount with permanent mounting medium. The Nissl body will be stained purple-blue.

After Nissl staining, neuronal cells in the hippocampal were identified from five sections (20 µm thickness, 80 µm apart) per rat (5 rats in each of Sev+N_2_O group, Con group, and Sev+N_2_O-MWM group, and 4 rats in Con-MWM group). In each section, the number of neurons was averaged from three random different vision fields in the hippocampal CA1 in each hemisphere (six vision fields per section) under Leica microscope (400× magnification). Only intact neurons with a clearly defined cell body and nucleus were counted.

### Double Labeling with NeuN and Annexin V

Apoptosis in hippocampus was detected using an Annexin V- FITC/neuronal marker NeuN doubled staining according to the protocol by Bian Z et al [34]. Brains were sectioned at 12 um thickness and mounted on cover slides followed by air-drying before labeling with Annexin V-FITC. Five Sections (60 µm space) per animal were stained first for Annexin V-FITC using an Annexin V staining Kit (BD Biosciences, CA) according to the manufacturer’s instructions, then blocked with blocking solution as described above, incubated with primary anti-NeuN antibody (1∶100, cell signaling) at RT for 2 h and secondary antibody for 1 h. Nuclear countstaining was performed with Hochest (Invitrogen, CA). Assessment of the doubled NeuN and Annexin V- positive neurons was performed in the CA1 subfield of the hippocampus.

### Nestin and DCX Immunohistochemistry

Nestin, a type VI intermediate filament protein, is predominantly expressed in neurogenic and myogenic stem cells, and has been known as a marker of neuroepithelial/progenitor cells. Doublecortin (DXC) is a microtubule-associated protein and has been known as a marker of immature neurons.

In this experiment, five sections (12 µm thickness, 60 µm space) per rat (n = 5 in each of Sev+N_2_O group, Con group, Sev+N_2_O-MWM group, and n = 4 in Con-MWM group) were analyzed. The protocol for Nestin and DCX staining was essentially the same as for pCREB staining, expect the primary antibody: anti-Nestin (1∶100, Boster, Wuhan, China) and anti-DCX (1∶100, Epitomics, Burlingame, CA). Analysis of the Nestin and DCX positive neurons was performed in the DG subfield of the hippocampus.

### Statistical Analysis

All analyses were performed using SPSS 18.0 for Windows (SPSS, Inc.). Data were submitted to repeated-measures ANOVA (the escape latencies and the swimming speed) and Tukey honestly significant difference test was used for post hoc testing in the Morris water maze. All the other analyses were conducted using Student unpaired two-tailed *t* test. A value of *p*<0.05 was considered significant.

## Results

### Aged Rats Exhibit Impaired Performance on Spatial Learning and Memory After Sevoflurane–N_2_O Anesthesia

No rat had an episode of hypoxia (defined as SpO_2_<90%) during the sevoflurane–N_2_O exposure, and rats did not exhibit floating behavior in our study.

**Figure 1:**
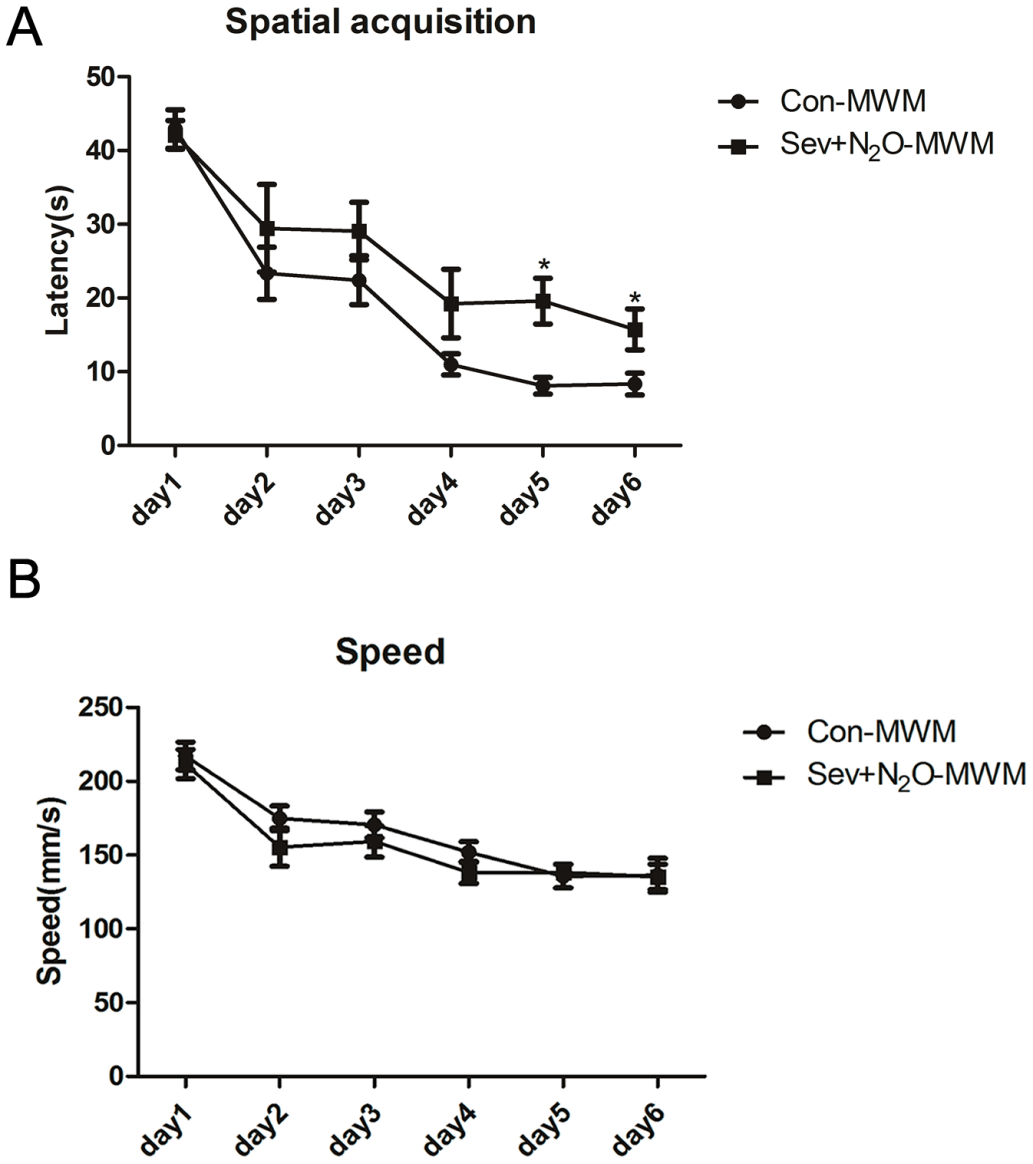
Swimming data for six consecutive training days in the Morris water maze. (A) The mean escape latency (in seconds) to find the hidden platform across training days. The significant difference were found on day 5 (*p* = 0.02) and day 6 (*p* = 0.024). (B) There was no difference in the swimming speed of the two groups during training days (*p* = 0.699). The data were analyzed by a repeated measure analysis of variance (ANOVA) and results are represented as mean ± SEM. *p<0.05.

Spatial acquisition for learning in the water maze is presented in Figure 1, which depicts the escape latency to reach the platform. In the first day of spatial acquisition trials, all rats tended to swim around the edges looking for an escape, but after being placed on the platform at the end of each trial, rats gradually learned that there was an escape platform and would venture into the center of the pool to locate it. By the second day’s session, all rats could rapidly locate the platform and the latency was significant less than the first day. A two-way repeated measure ANOVA on the escape latency revealed a significant effect of days for testing the water maze (*p*<0.001) and a main effect of group (*p* = 0.016), but no day × group interaction (*p* = 0.406; Figure 1A) and no effect of speed (*p* = 0.699; Figure 1B). In the Sev+N_2_O-MWM group, the impairment was found on day 5 (*p* = 0.02) and day 6 (*p* = 0.024), postanesthesia days 7 and 8, respectively, compared to the control rats, which represented the learning deficit after sevoflurane-N_2_O anesthesia. These results indicated that both the Con-MWM group and Sev+N_2_O-MWM group exhibited improvement in spatial learning and memory over time during the acquisition phase, but the latter had a longer latency to reach the target quadrant. The lack of effect of speed suggested that the poorer performance of the Sev+N_2_O-MWM group did not result from lack of motivation or reduced motor ability.

**Figure 2:**
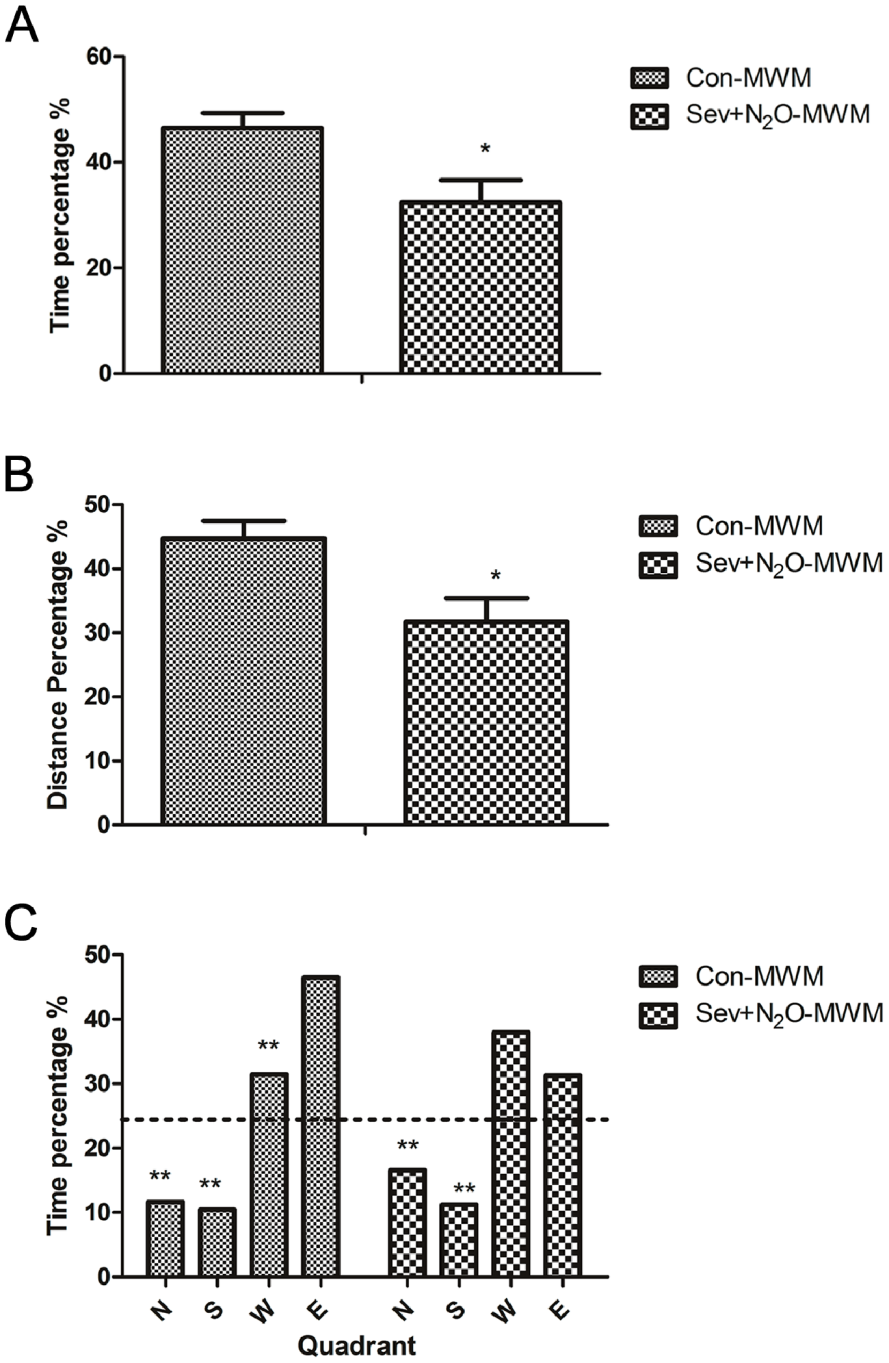
Probe trial conducted after the last water maze acquisition. (A) The percentage of time spent in the target quadrant. (B) The percentage of distance traveled in the target quadrant. (C) The percentage of time spent in each quadrant. Results are represented as mean ± SEM. *p<0.05, **p<0.01.

The probe trial conducted 24 h after the last water maze acquisition session was used to assess the spatial memory based on the percentage of time spent in the target quadrant (E quadrant) where platform had been located as well as the distance covered in the E quadrant (Figure 2). Figure 2A shows that Sev+N_2_O-MWM rats spent significantly less time in the E quadrant compared with Con-MWM rats (*p*<0.05), and Figure 2B shows that the distances travelled by Con-MWM rats in the E quadrant were more than those by Sev+N_2_O-MWM rats (*p*<0.05). Figure 2C shows that Con-MWM rats spent much more time in the E quadrant than the other three ones (all *p*<0.01), as revealed by a significant effect of quadrant (*p*<0.01). But unlike the Con-MWM group, the Sev+N_2_O-MWM rats spent no more time in the E quadrant than in the W quadrant (*p* = 0.381). Furthermore, *t*-test comparison confirmed that animals spent significantly more time in the target quadrant than would be expected by chance (*p*<0.01).

### The Expression of pCREB was Decreased in the Hippocampus of Aged Rats 48 h after Sevoflurane-N_2_O Anesthesia

**Figure 3:**
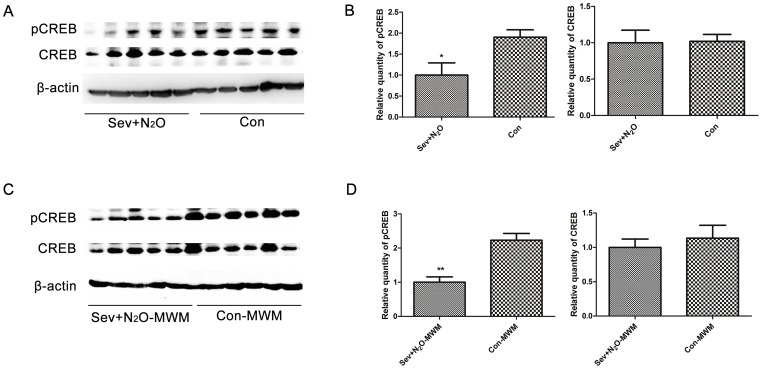
Phosphorylated CREB decreased 48-N_2_O anesthesia as well as 15 min after probe test. (A) The levels of pCREB in the Sev+N_2_O group were lower than the Con group, but no differences of CREB levels were detected. (B) Relative levels of pCREB and CREB were quantified (*p* = 0.029 for pCREB, *p* = 0.923 for CREB). (C) The pCREB levels in the hippocampus of Sev+N_2_O-MWM rats were significantly reduced compared to the Con-MWM rats, but CREB expression had no significant difference. (D) Relative levels of pCREB and CREB were quantified (*p* = 0.01 for pCREB, *p* = 0.58 for CREB). *p<0.05, **p<0.01.

**Figure 4:**
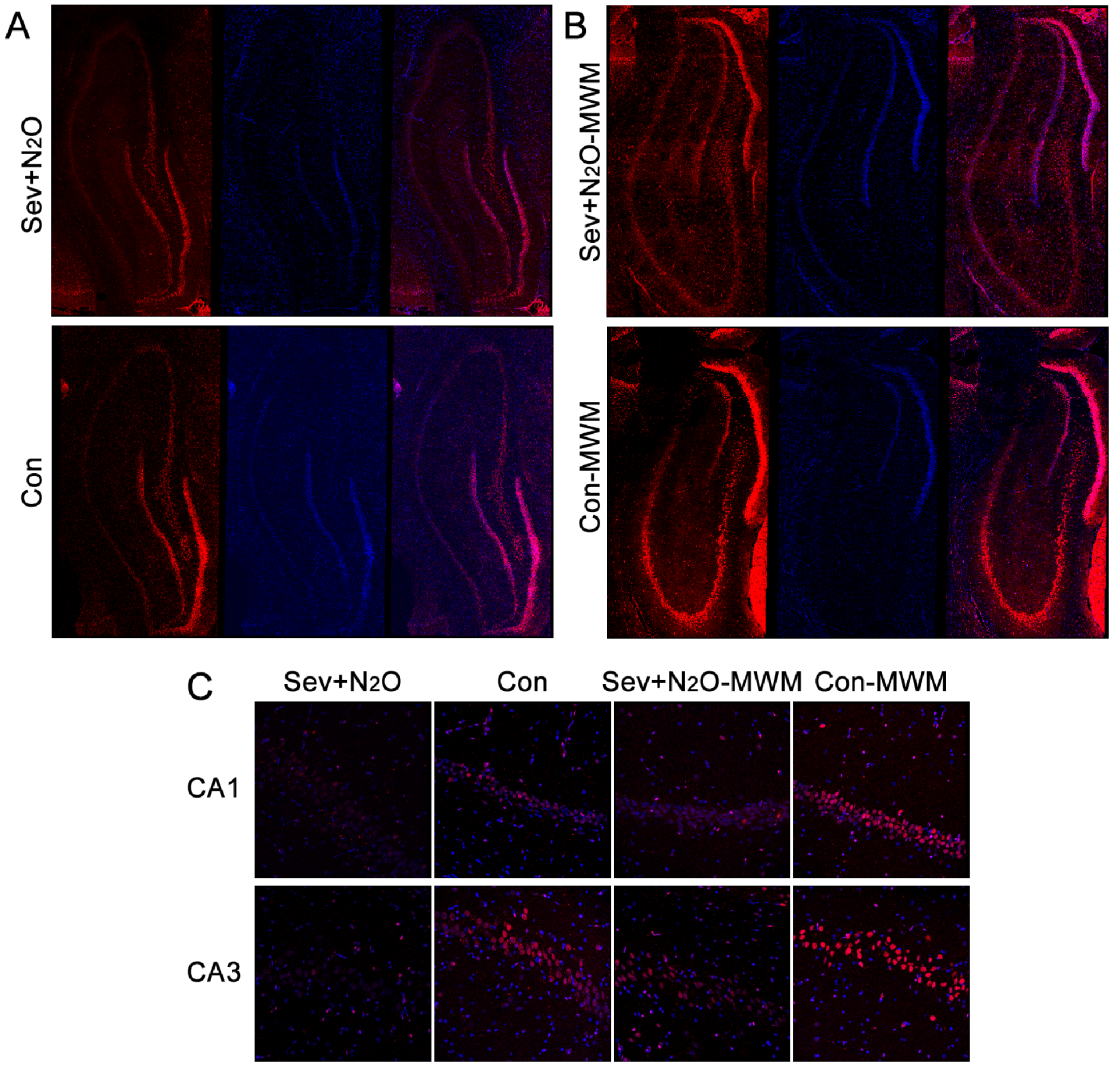
pCREB immunoreactivity (pCREB-ir) distribution in all the subfields of hippocampus and exclusively located in the neuronal nuclei. The pCREB-ir decreased both 48 h after sevoflurane-N_2_O anesthesia and 15 min after probe test. (A) There was a derease of pCREB-ir in all the subfields of the hippocampus in Sev+N_2_O rats compared with the Con group. (B) The pCREB-ir significantly decreased in the Sev+N2O-MWM group compared with Con-MWM group. (C) pCREB-ir both in CA1 and CA3 areas decreased in the exposed rats compared with controls. Magnification: ×200 for (A), ×400 for (B).

We killed the rats 48 h after anesthesia and measured the pCREB and total CREB levels in the Sev+N_2_O and Con rats. The Western blot results showed that the immunoreactive bands of pCREB as well as CREB appeared at 43 kDa, and the pCREB levels in the Sev+N_2_O group were significantly decreased (*p* = 0.029), but no significant difference in the levels of total CREB was observed (*p* = 0.923, Figure 3A, 3B). Figure 4A shows the pCREB immunoreactivity (pCREB-ir) distribution in all the subfields of the hippocampus, while Figure 4C shows the pCREB-ir in all the layers of CA1 and CA3 sections. The immunofluorescence revealed that pCREB was exclusively located in neuronal nuclei and there was a significant reduction of pCREB-ir in Sev+N_2_O rats compared with the Con group. These results implied that sevoflurane-N_2_O anesthesia would inhibit the activation of CREB 48 h after exposure in aged rats.

### Expression of pCREB in the Hippocampus of Aged Rats after Morris Water Maze

Both the Sev+N_2_O-MWM group and the control group were sacrificed 15 min after the probe trial on day7, and we compared the levels of pCREB between groups by Western blot. The results showed that the pCREB levels in the Sev+N_2_O-MWM rats were significant lower than those in the Con-MWM rats, but no significant difference of total CREB was detected (Figure 3C, 3D).

The immunofluorescence results showed that pCREB was higher in the Con-MWM group than in the Con group (Figure 4A, 4C), which was in accordance with our previous study showing that the retrieval of spatial memory could activate the phosphorylation of CREB [35]. However, the levels of pCREB in the Sev+N_2_O-MWM rats were significantly lower than those in the Con-MWM rats, suggesting that the decrease of pCREB expression induced by general anesthesia with Sev+N_2_O was sustained until at least the end of the behavioral procedures (Figure 4B, 4C).

### cAMP Levels

**Figure 5:**
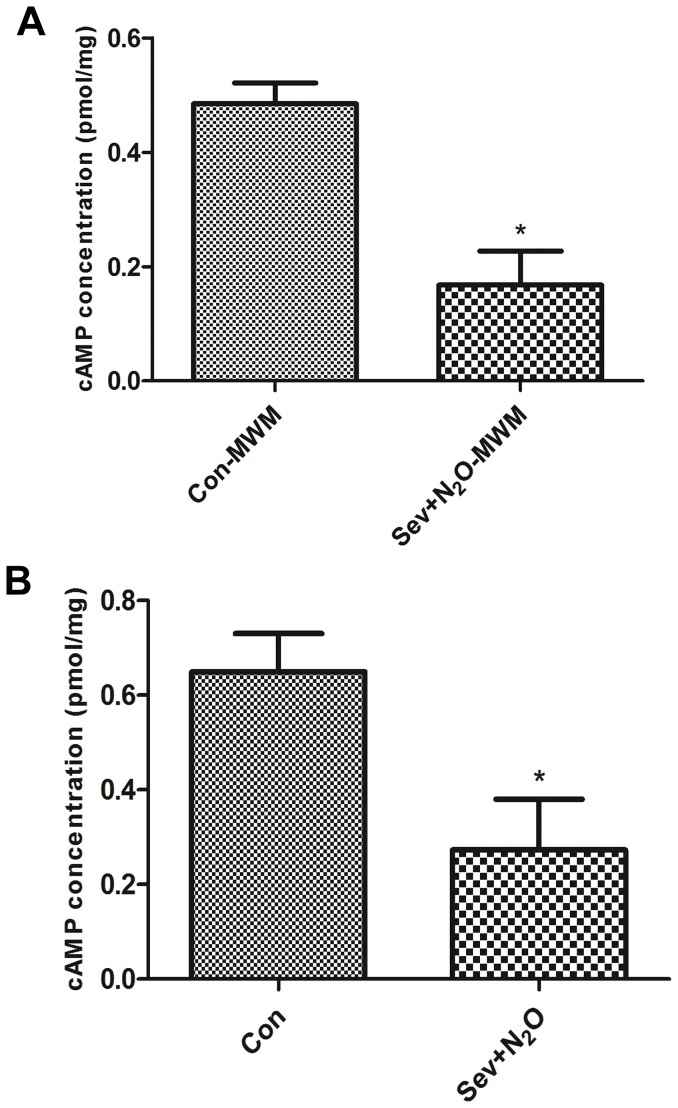
cAMP concentration in the hippocampus of all the groups. (A) cAMP levels decreased by 65% in hippocampus of Sev+N_2_O rats compared to the Con rats (*p* = 0.02). (B) cAMP levels decreased by 58% in Sev+N_2_O-MWM compared to Con-MWM (*p* = 0.048).

CREB phosphorylation can be achieved by a number of up-stream signaling cascades, among which a pathway is triggered by cAMP accumulation to cause liberation of catalytic subunits of cAMP – dependent protein kinase A (PKA) [36]. Because general anesthesia induced the decrease of pCREB expression, we assessed whether it was related to the cAMP pathway. The ELISA results revealed that the cAMP levels decreased by 65% in hippocampus of Sev+N_2_O rats compared to the Con rats (*p* = 0.02, Figure 5A). Similarly, in the Sev+N_2_O-MWM group, there was a 58% decrease of cAMP levels compared to Con-MWM (*p* = 0.048, Figure 5B). Thus, the results suggested that general anesthesia with Sev+N_2_O for 4 hours decreased the cAMP levels and in turn suppressed the cAMP/CREB signaling.

### Neuronal Cell Death Increased after Sev+N_2_O General Anesthesia

**Figure 6:**
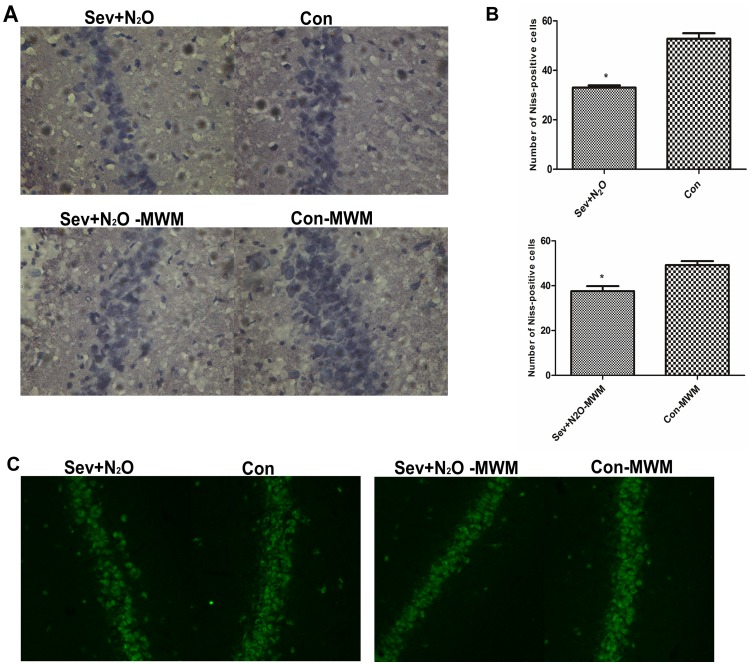
More neuronal loss in the CA1 subfield of hippocampus in exposed rats than control rats by Nissl staining and NeuN staing. (A) Nissl-positve cells in CA1 subfield decreased in the Sev+N_2_O group and Sev+N_2_O-MWM group compared with their respective controls. (B) The number of Nissl-postive cells was quantified. The number of neurons in each section was averaged from three random different vision fields in the CA1 area of hippocampus per hemisphere (six vision fields per section) under 40×objective Leica microscope. 5 sections per rat were analyzed (n = 5 in each of Sev+N_2_O, Con and Sev+N_2_O-MWM group, and n = 4 in Con-MWM group. *p* = 0.0286, 0.016, respectively). (C) Expression of the mature neuronal marker NeuN in CA1 subfield decreased in exposed rats compared with controls. Magnification: ×400 for (A), ×200 for (C).

**Figure 7:**
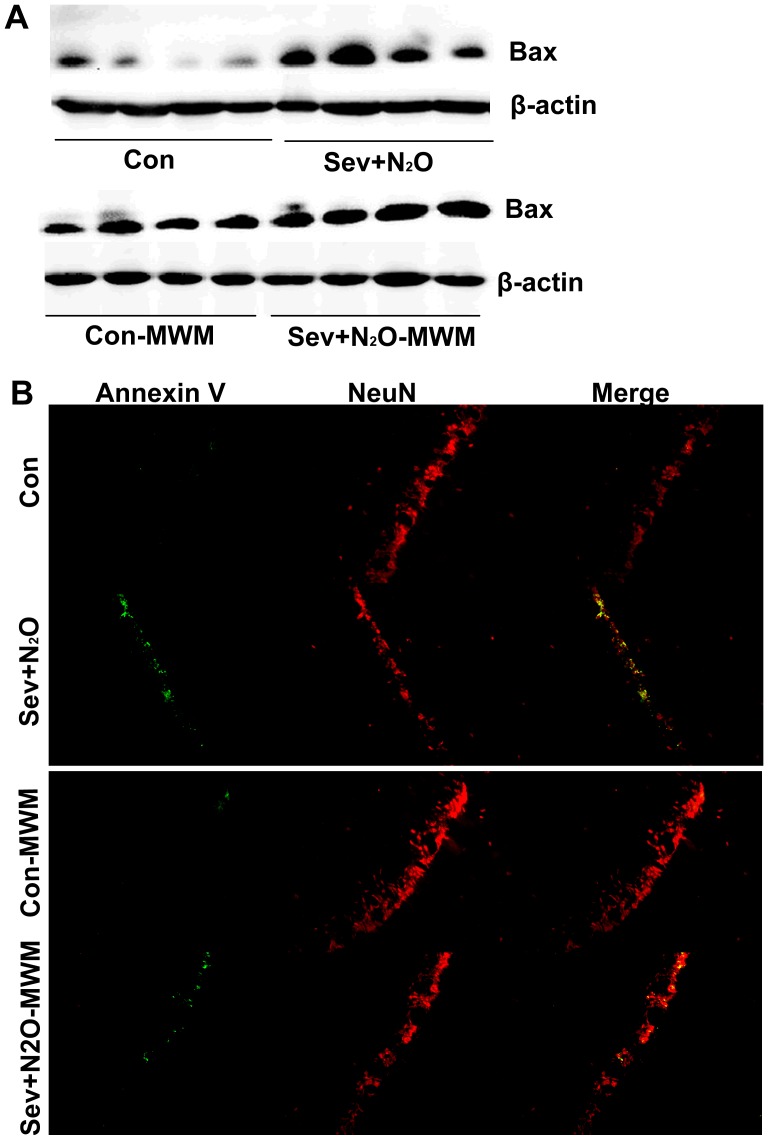
Expression of Bax was detected by Western Blot and neuronal apoptosis was evaluated by doubled staining with Annexin V-FITC and NeuN. (A) Bax expression was significantly increased in the Sev+N_2_O group compared with Con group, but only slightly increased in the Sev+N_2_O-MWM compared with Con-MWM group. (B) Doubled staining with Annexin V-FITC and NeuN showed that there were more neurons underwent apoptosis in the CA1 subfield of hippocampus in Sev+N_2_O and Sev+N_2_O-MWM rats. Magnification: ×200 for (B).

To understand whether cAMP/pCREB down-regulation affect the neuronal survival after Sev+N_2_O anesthesia, we evaluated the neuronal survival by Nissl staining and NeuN staining. Nissl staining is used for the detection of Nissl Body in the cytoplasm of neurons and identifying the basic neuronal structure. Quantitative analysis of Nissl-positive cells in hippocampal CA1 showed a neuronal loss in exposed rats compared with their controls (*p*<0.05, Figure 6A, B). Otherwise, expression of NeuN, a neuronal marker, also decreased in the Sev+N_2_O group as well as Sev+N_2_O-MWM group (Figure 6C). To confirm the neuronal loss is due to the neuronal apoptosis, double labeled of Annexin V-FITC and NeuN was performed. Figure 7B shows that the total number of Annexin V-positive cells in the hippocampal CA1 significantly increased in the rats exposed to anesthetics compared with the controls. However, most of the Annexin V-positive cells also stained positive for the neuronal marker NeuN. As loss of CREB triggered Bax-dependent apoptosis [31], it is possible that inhibition of CREB signaling by general anesthesia induces the Bax overexpression and contributes to the neuronal apoptosis. To verify it, we tested the Bax protein levels by Western Blot and found that the Bax expression significantly increased in the Sev+N_2_O group compared with Con group (Figure 7A). However, the Bax expression was only slightly higher in Sev+N_2_O-MWM rats than in Con-MWM rats (Figure 7A).

### Neurogenesis in the Hippocampal DG

**Figure 8:**
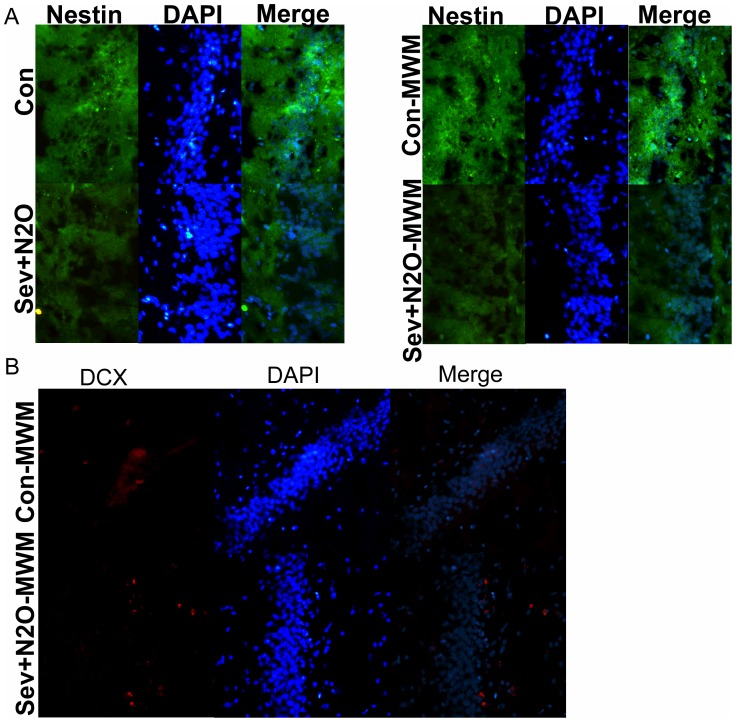
Expression of Nestin and DCX in DG area of hippocampus was detected by Immunofluorescence. (A) Nestin-postive cells were less in the exposed rats than controls. (B) DCX-positive cells decreased in the Sev+N_2_O-MWM group compared with Con-MWM group. Magnification: ×200 for (A) and (B).

As training on hippocampus-dependent tasks, such as the spatial Morris water maze, increases hippocampal neurogenesis [37]. Decreasing hippocampal neurogenesis has been demonstrated to impair long-term spatial memory [38]. We examined the possibility that the spatial learning and memory impairments in aged rats after general anesthesia with Sev+N_2_O might be related to a deficiency in DG neurogenesis due to the downregulation of cAMP/pCREB. We analyzed the differences in expression of various neuronspecific markers related to different neuronal stages, Nestin used as a marker of neuroepithelial/progenitor cells and DCX as immature neuronal marker. The immunofluorescence staining results showed that less neurons expressed Nestin in the Sev+N_2_O group as well as Sev+N_2_O-MWM group (Figure 8A). Moreover, we found less DCX-positive cells in the Sev+N_2_O-MWM group compared with Con-MWM group (Figure 8B). These results indicated that neuronal progenitor proliferation and differentiation was inhibited.

## Discussion

With the coming of the aging society, more and more surgical procedures currently performed are in elderly patients. Patients with an age≥60 years have an increased incidence of POCD, which is associated with an increased mortality [39], [40], [41]. The aged brain might be more susceptible to anesthetic-mediated changes, as the aged brain is different from the younger in several respects, including size, distribution and type of neurotransmitters, metabolic function, and capacity for plasticity [42]. As sevoflurane and N_2_O are commonly used anesthetic agents in clinical practice, the current study focused on the behavioral and biochemical effects to aged rats by general anesthesia with sevoflurane and N_2_O. We first found that 18-month-old rats developed cognitive deficits after exposure to 1.3% sevoflurane combined with 50% N_2_O for 4 h without surgery. It was unlikely that this impairment was caused by residual anesthetic because we did not perform the MWM training until 48 h after anesthesia. Culley et al [8] showed that aged rats subjected to ISO+N_2_O anesthesia exhibited poor performance on a spatial memory task for at least 2 weeks after general anesthesia, and the induced impairment may have been worse than ISO alone. Moreover, a 4-h exposure of aged rats to N_2_O alone has been reported to cause learning impairment up to 2 weeks [4]. Recent studies also showed that exposure to sevoflurane increased β-amyloid protein levels, which could induce further apoptosis and contribute to or cause POCD [1], [3], [43], [44], [45]. Sevoflurane at doses from 0.5% to 2.6% administered either during or immediately after a learning task has been shown to inhibit memory retention [46], [47], [48], [49], [50]. Although the inhalation anesthesia–POCD model has been well documented, it is debated. Rammes et al [51] reported ISO enhanced long-term potentiation (LTP) in CA1 hippocampal neurons and improved hippocampus-dependent cognitive performance, while a recent study demonstrated that 4 h of sevoflurane exposure did not impair acquisition learning or retention memory and might even improve learning in young adult or aged rat [52]. Different methodologies, including different anesthetic, dosage, rat strain, duration of exposure, outcome measurements, and anesthetic carrier gas may have contributed to these contradictory results. In the current study, we used 1.3% sevoflurane and 50% N_2_O as the anesthetics and 50% O_2_ for the carrier gas, which could be one of the factors that contributed to the differences.

The precise mechanism of molecular biology of POCD is still not very clear so far. Our current study tried to demonstrate the effects of anesthetics (i.e., sevoflurane, N_2_O) on biochemical changes associated with cognitive dysfunction. Current hypotheses attribute the neurocognitive deficits produced by anesthetics to neurotoxic effects, endogenous neurodegenerative, neuroinflammatory mechanisms, or age-sensitive suppression of neuronal stem cell proliferation and differentiation [53], [54], [55]. Moreover, brain-derived neurotrophic factor has been implicated in the neurotoxicity of N_2_O, midazolam, and ISO [56]. As mentioned previously, pCREB promotes the transcription of immediate-early gene mRNA, which is then translated into proteins. These proteins are necessary for the maintenance of LTP and long-term memory, while spatial learning has been shown to increase CREB phosphorylation in the dorsal hippocampus [12], [57], [58], [59], [60], [61]. In the current study, we measured the pCREB and CREB levels in the hippocampus 48 h after exposure but before water maze test, as well as 15 min after the probe trial. The results found that pCREB levels in the Sev+N_2_O and Sev+N_2_O-MWM group were significantly lower than their respective controls. Our results suggested that sevoflurane-N_2_O anesthesia decreased pCREB levels and lasted until at least the probe trial finished, which would affect the capacity of pCREB’s maintenance of LTP and long-term memory. However, we found the decrease of pCREB could be due to the phosphorylation of preexisting CREB, because there was no difference in total CREB levels among groups.

To identity the upstream regulators of CREB-signaling, studies have demonstrated that NMDA-receptor activation, BDNF (brain derived neurotrophic factor) signaling or growth factor signaling can trigger intracellular signaling cascades that phosphorylate CREB at Ser133, which is a rate-limiting step in the CREB-signaling [62], [63]. Among various signaling pathways, the most thoroughly pathway is to stimulate adenylyl cyclase and accumulate second messenger cAMP to activate PKA and lead to the release of the catalytical subunit of PKA which then shuttles to the nucleus and phosphorylates CREB [64]. In the current study, we found the cAMP levels decreased in the hippocampus of Sev+N_2_O group and Sev+N_2_O-MWM group compared to their respective control group. The above evidence indicated that general anesthesia with Sev+N_2_O down-regulated the cAMP/CREB signaling and further affected its downstream targets.

New neurons are produced each day through the process of neurogenesis in the hippocampus, while physical training can modify the process by increasing the number of new cells that mature into functional neurons in the adult brain and improve the cognitive ability including learning and memory [65]. In the past years, researchers have found that rats exposure to isoflurane on P7 showed decreased neuronal progenitor proliferation with deficits in fear conditioning and spatial reference task, and P14 rats exposed to isoflurane showed a decreased neurogenesis in hippocampus and impaired cognitive function compared with controls [66]. It has previously been noted that CREB signaling is essential for survival and morphological development of newborn neurons, and for maintenance of expression of proteins involved in neuronal development and the control of neurogenic transcriptional programs, while activation of cAMP signaling promotes the proliferation and morphological maturation of newborn cells [23], [24]. The in vivo function of CREB-signaling in adult neurogenesis has been examined using pharmacological, genetic and retrovirus-mediated gene transfer [67]. Thus, examination of DG neurogenesis may provide some information about how anesthetics cause learning and memory deficits via downregulation of CREB signaling. We found that the down-expression of pCREB in the hippocampus of rats exposed to Sev+N_2_O anesthesia disrupted the DG neurogenesis and exacerbated the cognitive dysfunction. This was underscored, at least in part, by our observation that the number of Nestin-positve cells and Dcx-positive cells was less in the exposed rats compared to the controls, which indicated the decrease of neuronal progenitor proliferation, differentiation and maturation.

Researchers have shown that CREB DNA binding activity and phosphorylation of CREB are necessary for nerve growth factor (NGF)-dependent survival of sympathetic neurons. Bonni et al [29] indicated that CREB may also have a function in the regulation of neuronal survival in the developing central nervous system. Furthermore, experiments using genetic transfer have demonstrated that CREB is a key executor of neurotrophin-mediated cell survival and loss of CREB triggers Bax-dependent apoptosis in vivo [31]. These findings demonstrated a critical role for CREB and CREB-dependent gene expression in supporting neuronal survival. In our work, we found an increase of neuronal loss in the exposed rats compared with controls. Annexin V-FITC by immunofluorescence and Bax expression by Westren blot revealed the neuronal loss was due to the trigger of neuronal apoptosis. Importantly, double staining of Annexin V and the neuronal marker NeuN indicated that most of the apoptotic cells in these structures were indeed neurons. In conjunction with the role of CREB in DG neurogenesis, these findings partly support our prediction that downregulation of cAMP/CREB induced by general anesthesia leads to a decrease of the neurogenesis in hippocampus as well as an increase of neuronal apoptosis, both of which play critical roles in the deficit of learning and memory after Sev+N_2_O exposure.

In summary, we found that general anesthesia with 1.3% sevoflurane and 50% N_2_O impaired hippocampus-dependent learning and memory in aged rats. To explore the mechanism of neurotoxicity induced by Sev+N_2_O, we found the downregulation of cAMP/CREB signaling which was implicated in the learning and memory, long term potentiation, and neuroprection. Furthermore, we observed the decrease of DG neurogenesis and neuronal survival in the hippocampus, both of which highly depended on the normal activation of CREB signaling. Thus, the pathway of cAMP/CREB-neurogensis/neuronal apoptosis contributed to the neurotoxicity and impairment of learning and memory induced by Sev+N_2_O. The study provided some theoretical basis for the further study of neurotrophin factors, which could prevent POCD in elderly patients and decrease the postoperative morbidity and mortality.
